# Joint Calibration Method for Robot Measurement Systems

**DOI:** 10.3390/s23177447

**Published:** 2023-08-26

**Authors:** Lei Wu, Xizhe Zang, Guanwen Ding, Chao Wang, Xuehe Zhang, Yubin Liu, Jie Zhao

**Affiliations:** State Key Laboratory of Robotics and Systems, Harbin Institute of Technology, Harbin 150001, China; wulei@stu.hit.edu.cn (L.W.); zangxizhe@hit.edu.cn (X.Z.); 17B908042@stu.hit.edu.cn (G.D.); wangchaohit@hit.edu.cn (C.W.); liuyubin@hit.edu.cn (Y.L.); jzhao@hit.edu.cn (J.Z.)

**Keywords:** joint calibration method, robot measurement system, binocular planar structured light camera, Lie algebra

## Abstract

Robot measurement systems with a binocular planar structured light camera (3D camera) installed on a robot end-effector are often used to measure workpieces’ shapes and positions. However, the measurement accuracy is jointly influenced by the robot kinematics, camera-to-robot installation, and 3D camera measurement errors. Incomplete calibration of these errors can result in inaccurate measurements. This paper proposes a joint calibration method considering these three error types to achieve overall calibration. In this method, error models of the robot kinematics and camera-to-robot installation are formulated using Lie algebra. Then, a pillow error model is proposed for the 3D camera based on its error distribution and measurement principle. These error models are combined to construct a joint model based on homogeneous transformation. Finally, the calibration problem is transformed into a stepwise optimization problem that minimizes the sum of the relative position error between the calibrator and robot, and analytical solutions for the calibration parameters are derived. Simulation and experiment results demonstrate that the joint calibration method effectively improves the measurement accuracy, reducing the mean positioning error from over 2.5228 mm to 0.2629 mm and the mean distance error from over 0.1488 mm to 0.1232 mm.

## 1. Introduction

Industrial robots are preferred for their high flexibility, low cost, and wide working range, and they have started to gradually replace manual labor in many scenarios, such as welding, shot peening, and palletizing [1,2,3]. A 3D camera utilizes the parallax principle to measure 3D coordinates, providing benefits such as high stability and measurement accuracy [4]. This type of camera is commonly mounted at the robot end-effector to form a measurement system for various applications, including reverse engineering and in-line inspection [5].

**Figure 1:**
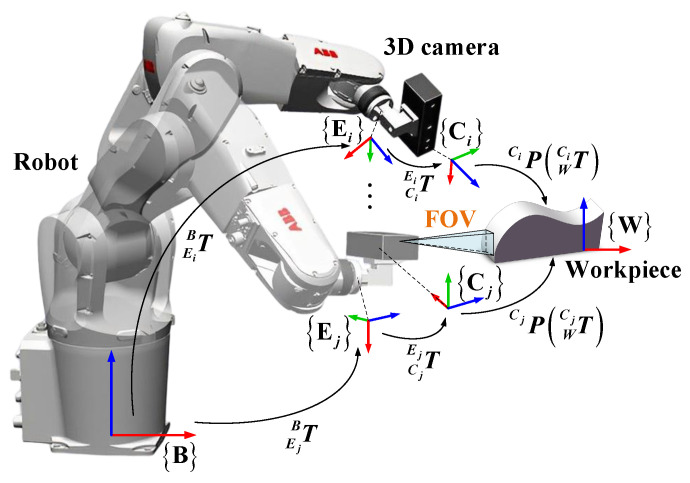
The robot measurement system.

The working process of a robot measurement system is illustrated in Figure 1. Due to the workpiece size often exceeding the camera’s field of view (FOV), local point clouds of the workpiece are captured from multiple sampled poses, and these point clouds are then transformed into the robot base coordinate system to generate a complete point cloud of the workpiece. The accuracy of the measurement system is jointly determined by the robot kinematics, camera-to-robot pose installation, and 3D camera measurement errors. Robot kinematic error is deviations in geometric parameters such as rod length and torsion angle, which causes errors in poses fed back by the robot controller [6]. Camera measurement error is influenced by the accuracy of the camera’s internal and external parameters, which are typically calibrated by the camera manufacturer [7]. However, secondary calibration of the camera can improve its measurement accuracy [8]. The relative pose between the camera and robot is typically achieved through hand–eye calibration, and its accuracy is impacted by both robot kinematic error and camera measurement error [9]. A large measurement error can result in inaccurate measurement models and low-quality products, making it crucial to calibrate these errors before using the measurement system. These three types of errors are referred to simply as robot error, hand–eye matrix error, and camera error in this paper.

Some achievements have been made in the separate calibration of each error. The D-H method, screw method, and Lie algebra method are often used to model robot error [10,11,12]. The D-H method is known to have singularities and discontinuity in its parameters, whereas the screw method has a systematic associated error [13]. These problems are not present in the Lie algebra method. In addition, external equipment or calibrators are often used to provide high-accuracy raw data, and constraint equations are established to solve for kinematic errors [14,15]. Calibration methods that rely on calibrators tend to be simpler. Hand–eye calibration for 3D cameras has not been sufficiently studied. Due to their limited precision for measuring jumping edges and vertices [16], calibration methods often favor the use of calibrators featuring circular elements such as spheres, discs, and holes [17,18,19,20]. However, most hand–eye calibration methods do not consider robot and camera errors, which influence calibration accuracy. For camera error, many studies have investigated the relationship between camera parameters and measurement error [21,22,23]. However, these methods are not suitable for users who lack knowledge of the camera parameters. Other studies have focused on establishing the relationship between the measurement value and measurement error [8,24,25]. However, these error models are proposed entirely based on observations and experience, without considering the measurement principle of 3D cameras.

It is crucial to highlight that separate error calibration is inadequate in ensuring the accuracy of the measurement system. Instead, a joint calibration method that considers robot, hand–eye matrix, and camera errors is necessary. Few researchers have paid attention to this aspect. Some studies established joint error models based on the D-H method and used geometric constraints to calibrate robot and hand–eye matrix errors simultaneously [26,27]. However, the calibration results of these methods are unstable due to the drawbacks of the D-H method. To address this issue, Lie algebra has been used to establish a joint error model, resulting in improved stability [13]. However, the absolute position data were used for calibration in the study, which requires the use of high-precision external equipment. In addition, none of these studies account for camera error, which can affect the accuracy of the joint calibration.

We thoroughly consider the advantages and limitations of previous studies and propose a joint calibration approach that takes into account three types of error. A standard sphere is used as the calibrator considering the features of the 3D camera, and the sphere center is captured as the raw data for calibration. Separate models are established for each type of error. The error models for the robot and hand–eye matrix are established using Lie algebra, whereas a pillow model is created for the camera based on its error distribution and measurement principles. These error models are then integrated to construct the joint error model, which is used to establish a stepwise optimization problem for joint error calibration. Our contribution can be concluded as follows.

(1) The camera error, which is proved to influence the calibration accuracy in the simulation, is taken into account during joint calibration for the first time.

(2) A camera error model is established based on the measurement principle and error distribution, whereas a joint error model is established for joint calibration.

(3) The calibration problem is transformed into an optimization problem that minimizes the sum of the relative position error between the calibrator and robot, avoiding reliance on external equipment.

## 2. Method

### 2.1. Problem Statement

The typical robot measurement system is shown in Figure 1, with a 3D camera installed at the robot end-effector. In this system, several coordinate systems are defined. The robot base coordinate system is denoted as {B}, the robot end coordinate system as {E}, the camera coordinate system as {C}, and the workpiece coordinate system as {W}, which has the same pose as {B}, as shown in Figure 1. The transformation relationships between these coordinate systems are represented by homogeneous matrices. For example, ${}_{E}^{B}T$ represents the pose of {E} in {B}, or the transformation from {B} to {E}. During the measurement procedure, the point cloud ${}^{C}P$ in {C} needs to be transformed into {B} using ${}^{B}P={}_{E}^{B}T{}_{C}^{E}T{}^{C}P$. The errors in ${}_{E}^{B}T$, ${}_{C}^{E}T$, and ${}^{C}P$ are the robot, hand–eye matrix, and camera errors mentioned in Section 1. According to ${}^{B}P={}_{E}^{B}T{}_{C}^{E}T{}^{C}P$, there are also error in ${}^{B}P$, which is referred to as compensative error in this paper, and the accuracy of ${}^{B}P$ determines the accuracy of the measurement system.

This paper focuses on a joint calibration method for the robot, hand–eye matrix, and camera errors. Separate error models and the joint error model are first proposed. Then, the calibration problem is transformed into a stepwise optimization problem, and analytical solutions for each calibration parameter are derived.

### 2.2. Joint Calibration Method

**Figure 2:**
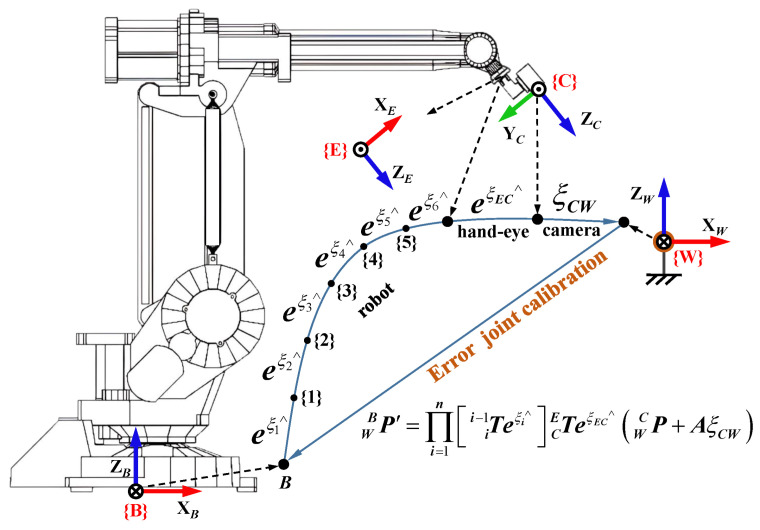
The joint calibration method.

The principle of the joint calibration method is depicted in Figure 2. A ceramic standard sphere is utilized as the calibrator and fixed at an appropriate position. The calibrator coordinate system is also donated as {W}, and the sphere center as W. Local point clouds of the sphere are collected from various sampling poses, and then spherical fitting is performed to obtain ${}_{W}^{C}P$, which represents the position of the sphere center W in {C}. As the relative position between the calibrator and robot is fixed, ${}_{W}^{B}P$ should always be equal to its ideal value ${}_{W}^{B}P_{0}$. However, ${}_{W}^{B}P={}_{E}^{B}T{}_{C}^{E}T{}_{W}^{C}P\neq {}_{W}^{B}P_{0}$ occurs due to errors in ${}_{E}^{B}T$, ${}_{C}^{E}T$, and ${}_{W}^{C}P$. The joint calibration method is achieved by calibrating ${}_{W}^{B}P$, and the specific principle is as follows.

Firstly, the robot error is modeled. ${}_{E}^{B}T$ can be represented as ${}_{E}^{B}T=\prod_{i=1}^{n}{}_{i}^{i-1}T$ using the modified Denavit–Hartenberg (MDH) method [28], where ${}_{i}^{i-1}T$ is the transformation matrix from the (i−1)th joint coordinate system to the *i*th joint coordinate system. The transformation matrix ${}_{i}^{i-1}T$ is calibrated by right-multiplying the calibration matrix $E_{i}$. $E_{i}$ can be represented by Lie algebra $\xi_{i}$ as $E_{i}=e^{\xi_{i}^{\wedge }}$, where $\xi_{i}={\left[\rho_{i},\phi_{i}\right]}^{T}\in se(3)$. The symbol ∧ denotes the skew-symmetric matrix, as shown in Equation (1), and the expression for $\xi_{i}^{\wedge }$ is $\left[\begin{array}{cc} \phi_{i}^{\wedge } & \rho_{i}\\ 0^{T} & 0 \end{array}\right]$. Then, the calibration value ${}_{E}^{B}T^{\prime }$ of ${}_{E}^{B}T$ can be obtained as shown in Equation (2).

(1) $$v^{\wedge }={\left[\begin{array}{c} v_{1}\\ v_{2}\\ v_{3} \end{array}\right]}^{\wedge }=\left[\begin{array}{ccc} 0 & -v_{3} & v_{2}\\ v_{3} & 0 & -v_{1}\\ -v_{2} & v_{1} & 0 \end{array}\right]$$

(2) $${}_{E}^{B}T^{\prime }=\prod_{i=1}^{n}\left[{}_{i}^{i-1}TE_{i}\right]=\prod_{i=1}^{n}\left[{}_{i}^{i-1}Te^{{\xi_{i}}^{\wedge }}\right]$$

The hand–eye matrix ${}_{C}^{E}T$ is likewise calibrated using the calibration matrix $E_{CE}$ and Lie algebra $\xi_{CE}$, as shown in Equation (3).

(3) $${}_{C}^{E}T^{\prime }={}_{C}^{E}TE_{EC}={}_{C}^{E}Te^{{\xi_{EC}}^{\wedge }}$$

**Figure 3:**
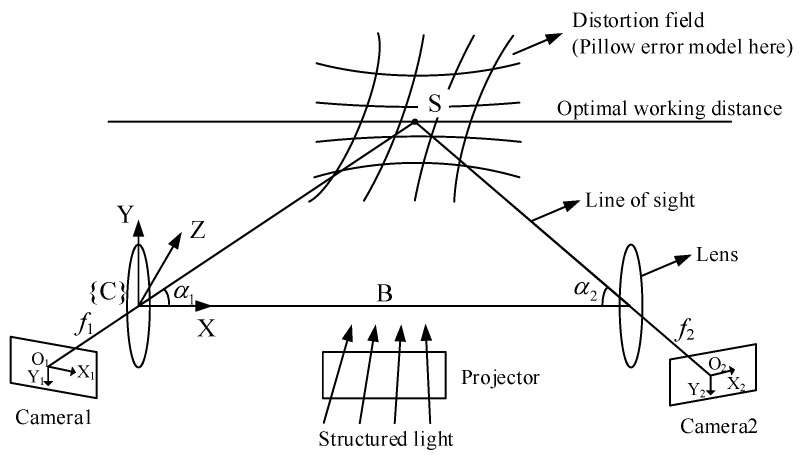
The measurement principle and error model of the 3D camera.

Finally, the camera error is modeled. The measurement principle of the 3D camera is illustrated in Figure 3. The structured light is projected onto the object, and two cameras simultaneously capture patterns on the object’s surface. Then, x-axis and z-axis coordinates are obtained based on the similar triangle relationship in the XOZ plane, followed by the computation of the y-axis coordinate through the geometric relationship. The coordinate accuracy depends on the accuracy of the baseline *B*, tilt angles $\alpha_{1},\;\alpha_{2}$, focal lengths $f_{1},\;f_{2}$, and image resolutions $X_{1},\;X_{2},\;Y_{1},\;Y_{2}$. However, these parameters are coupled, leading to a complex error form that is difficult to model and identify. To simplify, we will establish an error model that relates the error to the measurement value. In addition, the error model should consider the following characteristics based on the measurement principle and existing studies [21,22,29,30]. (1) The error increases as the measurement point moves away from point S, which is the intersection point of the two cameras’ lines of sight. This indicates the presence of a distortion field around S, as illustrated in Figure 3. (2) The error in the z-axis coordinate is generally larger than those of the x-axis and y-axis coordinates. (3) Different cameras may exhibit various forms of error.

**Figure 4:**
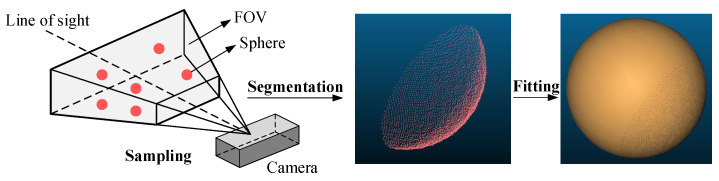
The observation process of the camera error distribution.

The Surface HD50 camera is used in this study, with a repeatability accuracy of ±0.15 mm, an optimal working distance of 500 ± 250 mm, and an FOV of H55° × V36°. To observe the camera’s error distribution, a ceramic standard sphere with a diameter of 38.1 mm and a diameter deviation of no more than 1 µm is placed within the camera’s 250–400 mm working distance. The fitted diameter is compared with the theoretical diameter to observe the camera’s error. This process is illustrated in Figure 4.

**Figure 5:**
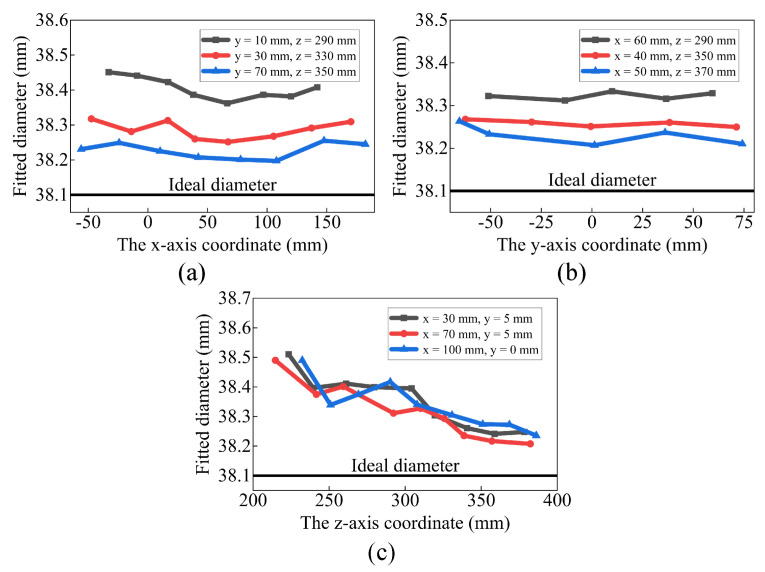
The camera error distribution (**a**) along the x-axis direction, (**b**) along the y-axis direction, and (**c**) along the z-axis direction.

Figure 5 displays the fitted diameter of the measurement points, where the horizontal coordinate represents the positions of the measurement points and the vertical coordinate represents the fitted diameter. Several patterns can be observed in Figure 5. (1) The fitted diameter of all measurement points is larger than the theoretical diameter. (2) The fitted diameter decreases obviously as the z-axis coordinate increases. (3) The fitted diameter tends to decrease as the x-axis coordinate increases, followed by a slight increase. (4) The fitted diameter is not related to the y-axis coordinate. These patterns suggest the existence of a pillow error at point S, causing the fitted diameter to increase as the measurement point moves away from S, as illustrated in Figure 3. Referring to the pillow distortion of color cameras, the error model of the 3D camera is defined as Equation (4). This expression takes into account the measurement principle of the camera, which states that the x-axis and z-axis coordinates are independent of the y-axis coordinate.

(4) $$\left\{\begin{array}{c} \Delta x=\sum_{i=1}^{m}k_{i}\left(x-x_{s}\right){\left[{\left(x-x_{s}\right)}^{2}+{\left(z-z_{s}\right)}^{2}\right]}^{i}\\ \Delta y=\sum_{j=1}^{m}k_{j}\left(y-y_{s}\right){\left[{\left(x-x_{s}\right)}^{2}+{\left(y-y_{s}\right)}^{2}+{\left(z-z_{s}\right)}^{2}\right]}^{j}\\ \Delta z=\sum_{l=1}^{m}k_{l}\left(z-z_{s}\right){\left[{\left(x-x_{s}\right)}^{2}+{\left(z-z_{s}\right)}^{2}\right]}^{l} \end{array}\right.$$

In Equation (4), $\left(\Delta x,\Delta y,\Delta z\right)$, $\left(x,y,z\right)$, and $\left(x_{s},y_{s},z_{s}\right)$ represent the calibration value, the measurement value, and the coordinate of point S, respectively. $k_{i}$, $k_{j}$, and $k_{l}$ are the calibration parameters, whereas *i*, *j*, *l*, and *m* are positive integers. The coordinate of point S for the used camera is theoretically $\left(60\;\mathrm{mm},0\;\mathrm{mm},500\;\mathrm{mm}\right)$. Based on the second error characteristic of the camera, the errors in the x-axis and y-axis coordinates can be neglected. Furthermore, m=3 is set to simplify the expression, and thus, the error model can be expressed as Equation (5), where $a_{i}=\left(z-z_{s}\right){\left[{\left(x-x_{s}\right)}^{2}+{\left(z-z_{s}\right)}^{2}\right]}^{i},\;i=1,2,3$.

(5) $${}_{W}^{C}P^{\prime }={}_{W}^{C}P+\underset{A}{\underbrace{\left[\begin{array}{ccc} 0 & 0 & 0\\ 0 & 0 & 0\\ a_{1} & a_{2} & a_{3}\\ 0 & 0 & 0 \end{array}\right]}}\underset{\xi_{CW}}{\underbrace{\left[\begin{array}{c} k_{1}\\ k_{2}\\ k_{3} \end{array}\right]}}={}_{W}^{C}P+A\xi_{CW}$$

The calibration value ${}_{W}^{B}P^{\prime }$, as shown in Equation (6), can be obtained by combining these separate error models. Theoretically, ${}_{W}^{B}P^{\prime }$ should be equal to ${}_{W}^{B}P_{0}$.

(6) $${}_{W}^{B}P^{\prime }=\prod_{i=1}^{n}\left[{}_{i}^{i-1}Te^{{\xi_{i}}^{\wedge }}\right]{}_{C}^{E}Te^{{\xi_{EC}}^{\wedge }}\left({}_{W}^{C}P+A\xi_{CW}\right)$$

${}_{W}^{B}P_{1}^{\prime },{}_{W}^{B}P_{2}^{\prime }\cdots {}_{W}^{B}P_{m}^{\prime }$ can be obtained from *m* sampling poses, and Equation (7) is obtained by taking pairwise differences between ${}_{W}^{B}P_{1}^{\prime },{}_{W}^{B}P_{2}^{\prime }\cdots {}_{W}^{B}P_{m}^{\prime }$.

(7) $$\left\{\begin{array}{c} \begin{array}{c} {}_{W}^{B}{P^{\prime }}_{1}-{}_{W}^{B}{P^{\prime }}_{2}=\\ \prod_{i=1}^{n}\left[{}_{i}^{i-1}T_{1}e^{{\xi_{i}}^{\wedge }}\right]{}_{C}^{E}Te^{{\xi_{EC}}^{\wedge }}\left({}_{W}^{C}P_{1}+A_{1}\xi_{CW}\right)-\\ \prod_{i=1}^{n}\left[{}_{i}^{i-1}T_{2}e^{{\xi_{i}}^{\wedge }}\right]{}_{C}^{E}Te^{{\xi_{EC}}^{\wedge }}\left({}_{W}^{C}P_{2}+A_{2}\xi_{CW}\right) \end{array}\\ \;\;\;\vdots \\ \begin{array}{c} {}_{W}^{B}{P^{\prime }}_{m-1}-{}_{W}^{B}{P^{\prime }}_{m}=\\ \prod_{i=1}^{n}\left[{}_{i}^{i-1}T_{m-1}e^{{\xi_{i}}^{\wedge }}\right]{}_{C}^{E}Te^{{\xi_{EC}}^{\wedge }}\left({}_{W}^{C}P_{m-1}+A_{m-1}\xi_{CW}\right)-\\ \prod_{i=1}^{n}\left[{}_{i}^{i-1}T_{m}e^{{\xi_{i}}^{\wedge }}\right]{}_{C}^{E}Te^{{\xi_{EC}}^{\wedge }}\left({}_{W}^{C}P_{m}+A_{m}\xi_{CW}\right) \end{array} \end{array}\right.$$

Since the relative position of the calibrator and robot is fixed, the equations in Equation (7) should theoretically be equal to 0. Equation (7) is then transformed into an optimization problem, as shown in Equation (8), where $x\left(\xi_{i}\left(i=1,2,\cdots ,6\right),\xi_{EC},\xi_{CW}\right)$ is to be solved.

(8) $$\begin{array}{c} \begin{array}{c} \min_{x}f\left(x\right)=\min_{x}\frac{1}{2}\sum_{j=2}^{m}∥\prod_{i=1}^{n}\left[{}_{i}^{i-1}T_{j-1}e^{{\xi_{i}}^{\wedge }}\right]{}_{C}^{E}Te^{{\xi_{EC}}^{\wedge }}\left({}_{W}^{C}P_{j-1}\right.+\\ \left.A_{j-1}\xi_{CW}\right){-\prod_{i=1}^{n}\left[{}_{i}^{i-1}T_{j}e^{{\xi_{i}}^{\wedge }}\right]{}_{C}^{E}Te^{{\xi_{EC}}^{\wedge }}\left({}_{W}^{C}P_{j}+A_{j}\xi_{CW}\right)∥}^{2} \end{array} \end{array}$$

$\xi_{i}\left(i=1,2,\cdots ,6\right)$, $\xi_{EC}$, and $\xi_{CW}$ are independent of each other, allowing them to be solved step by step. The process of solving $\xi_{k}$ is as follows. Let $B_{k}=\prod_{i=1}^{k-1}\left[{}_{i}^{i-1}Te^{{\xi_{i}}^{\wedge }}\right]{}_{k}^{k-1}T$ and $C_{k}=\prod_{i=k+1}^{n}\left[{}_{i}^{i-1}Te^{{\xi_{i}}^{\wedge }}\right]{}_{C}^{E}Te^{{\xi_{EC}}^{\wedge }}\left({}_{W}^{C}P+A\xi_{CW}\right)$, and define $\delta \xi_{k}$ to update $\xi_{k}$ in each iteration, with the update expression given by $e^{{\xi_{k}}^{\wedge }}=e^{\delta {\xi_{k}}^{\wedge }}e^{{\xi_{k}}^{\wedge }}$. Then, Equation (8) can be transformed into Equation (9).

(9) $$\begin{array}{c} \min_{E_{k}}f\left(E_{k}\right)=\min_{E_{k}}\frac{1}{2}\sum_{j=2}^{m}{∥B_{kj-1}e^{\delta {\xi_{k}}^{\wedge }}e^{{\xi_{k}}^{\wedge }}C_{kj-1}-B_{kj}e^{\delta {\xi_{k}}^{\wedge }}e^{{\xi_{k}}^{\wedge }}C_{kj}∥}^{2} \end{array}$$

Define $g_{j-1}\left(\delta \xi_{k}\right)$ as $g_{j-1}\left(\delta \xi_{k}\right)=B_{kj-1}e^{\delta {\xi_{k}}^{\wedge }}e^{{\xi_{k}}^{\wedge }}C_{kj-1}-B_{kj}e^{\delta {\xi_{k}}^{\wedge }}e^{{\xi_{k}}^{\wedge }}C_{kj}$. Since $\delta \xi_{k}$ is small, $g_{j-1}\left(\delta \xi_{k}\right)$ can be simplified using the first-order Taylor expansion around $\delta \xi_{k}=0$, yielding $g_{j-1}\left(\delta \xi_{k}\right)=B_{kj-1}e^{{\xi_{k}}^{\wedge }}C_{kj-1}-B_{kj}e^{{\xi_{k}}^{\wedge }}C_{kj}+\left(B_{kj-1}\frac{\partial e^{{\xi_{k}}^{\wedge }}C_{kj-1}}{\partial \delta \xi_{k}}-B_{kj}\frac{\partial e^{{\xi_{k}}^{\wedge }}C_{kj}}{\partial \delta \xi_{k}}\right)\delta \xi_{k}$. Equation (10) is obtained by substituting the simplified $g_{j-1}\left(\delta \xi_{k}\right)$ into Equation (9).

(10) $$\min_{\delta \xi_{k}}f\left(\delta \xi_{k}\right)=\min_{\delta \xi_{k}}\frac{1}{2}\sum_{j=2}^{m}\left[g_{j-1}{\left(\delta \xi_{k}\right)}^{T}g_{j-1}\left(\delta \xi_{k}\right)\right]$$

Define $h_{j-1}=B_{kj-1}\frac{\partial e^{{\xi_{k}}^{\wedge }}C_{kj-1}}{\partial \delta \xi_{k}}-B_{kj}\frac{\partial e^{{\xi_{k}}^{\wedge }}C_{kj}}{\partial \delta \xi_{k}}$, $p_{j-1}=B_{kj-1}e^{{\xi_{k}}^{\wedge }}C_{kj-1}-B_{kj}e^{{\xi_{k}}^{\wedge }}C_{kj}$, and $\delta \xi_{k}$ can be obtained as Equation (11) by solving the partial derivative $\frac{\partial f\left(\delta \xi_{k}\right)}{\partial \delta \xi_{k}}=0$.

(11) $$\delta \xi_{k}=-{\left[\sum_{j=2}^{m}\left({h_{j-1}}^{T}h_{j-1}\right)\right]}^{-1}\left[\sum_{j=2}^{m}\left({h_{j-1}}^{T}p_{j-1}\right)\right]$$

$\frac{\partial e^{{\xi_{k}}^{\wedge }}C_{kj-1}}{\partial \delta \xi_{k}}$ and $\frac{\partial e^{{\xi_{k}}^{\wedge }}C_{kj}}{\partial \delta \xi_{k}}$ can be derived using the definition of the derivative, as shown in Equations (12) and (13), where the symbol ∧ denotes the skew-symmetric matrix. *R* and *t* represent the rotation matrix and translation vector of $e^{{\xi_{k}}^{\wedge }}$.

(12) $$\frac{\partial e^{{\xi_{k}}^{\wedge }}C_{kj-1}}{\partial \delta \xi_{k}}=\left[\begin{array}{cc} I & -{\left(RC_{kj-1}+t\right)}^{\wedge }\\ 0^{T} & 0 \end{array}\right]$$

(13) $$\frac{\partial e^{{\xi_{k}}^{\wedge }}C_{kj}}{\partial \delta \xi_{k}}=\left[\begin{array}{cc} I & -{\left(RC_{kj}+t\right)}^{\wedge }\\ 0^{T} & 0 \end{array}\right]$$

Then, the hand–eye matrix calibration parameter $\xi_{EC}$ is derived. By defining $D=\prod_{i=1}^{n}\left[{}_{i}^{i-1}Te^{{\xi_{i}}^{\wedge }}\right]{}_{C}^{E}T$ and $F=\left({}_{W}^{C}P+\xi_{CW}\right)$, and using $\delta \xi_{EC}$ to update $\xi_{EC}$, the optimization problem $\min_{\delta \xi_{EC}}f\left(\delta \xi_{EC}\right)=\min_{\delta \xi_{EC}}\frac{1}{2}\sum_{j=2}^{m}{∥D_{j-1}e^{\delta {\xi_{EC}}^{\wedge }}e^{{\xi_{EC}}^{\wedge }}F_{j-1}-D_{j}e^{\delta {\xi_{EC}}^{\wedge }}e^{{\xi_{EC}}^{\wedge }}F_{j}∥}^{2}$ can be formulated. $\delta \xi_{EC}$ can be obtained following the similar derivation as Equations (9)–(13).

Finally, $\xi_{CW}$ is derived. Let $G=\prod_{i=1}^{n}\left[{}_{i}^{i-1}Te^{{\xi_{i}}^{\wedge }}\right]{}_{C}^{E}Te^{{\xi_{EC}}^{\wedge }}\left({}_{W}^{C}P+A\xi_{CW}\right)$ and $H=\prod_{i=1}^{n}\left[{}_{i}^{i-1}Te^{{\xi_{i}}^{\wedge }}\right]{}_{C}^{E}Te^{{\xi_{EC}}^{\wedge }}A$, and define $\delta \xi_{CW}$ to update $\xi_{CW}$, with the expression given by $\xi_{CW}=\xi_{CW}+\delta \xi_{CW}$. Then, Equation (8) can be transformed into Equation (14).

(14) $$\begin{array}{c} \min_{\delta \xi_{CW}}f\left(\delta \xi_{CW}\right)=\min_{\delta \xi_{CW}}\frac{1}{2}\sum_{j=2}^{m}{∥G_{j-1}-G_{j}+\left(H_{j-1}-H_{j}\right)\delta \xi_{CW}∥}^{2} \end{array}$$

$\delta \xi_{CW}$ can be obtained as Equation (15) by solving the partial derivative $\frac{\partial f\left(\delta \xi_{CW}\right)}{\partial \delta \xi_{CW}}=0$.

(15) $$\begin{array}{c} \delta \xi_{CW}=-{\left[\sum_{j=2}^{m}{\left(H_{j-1}-H_{j}\right)}^{T}\left(H_{j-1}-H_{j}\right)\right]}^{-1}\left[\sum_{j=2}^{m}{\left(H_{j-1}-H_{j}\right)}^{T}\left(G_{j-1}-G_{j}\right)\right] \end{array}$$

In each iteration, the calibration parameter *x* is calculated, and the objective function value $f\left(x\right)$ is updated by substituting *x* into Equation (8). The iterative process continues until $f\left(x\right)$ no longer decreases or the maximum number of iterations *L* has been reached. Algorithm 1 presents the pseudo-code for the iterative process. The initial ${}_{C}^{E}T$ is obtained using a calibration method described in Reference [26].   
**Algorithm 1:** The pseudo-code of calibration process
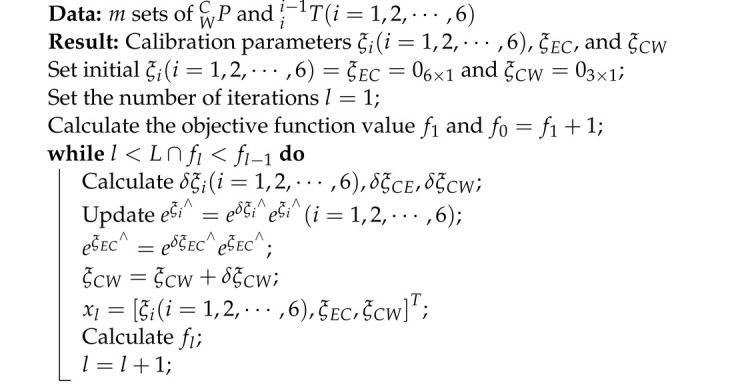


## 3. Simulation

The simulation is designed to verify the joint calibration method. Firstly, the simulation data are generated. The robot is modeled using the MDH method with parameter errors to generate ${}_{E}^{B}T$, and the pillow error model, as shown in Equation (5), is introduced to generate ${}_{W}^{C}P$. Furthermore, random noise is added to ${}_{E}^{B}T$ and ${}_{W}^{C}P$. Then, ${}_{E}^{B}T$, ${}_{C}^{E}T$ and ${}_{W}^{C}P$ are substituted into Algorithm 1 to obtain *x*, and calibrated values ${}_{W}^{B}P_{\mathrm{calibrated}}$, ${}_{E}^{B}T_{\mathrm{calibrated}}$, ${}_{C}^{E}T_{\mathrm{calibrated}}$, and ${}_{W}^{C}P_{\mathrm{calibrated}}$ are calculated according to *x*. Finally, the performance of the joint calibration method is evaluated by comparing the calibrated values with ideal values ${}_{W}^{B}P_{0}$, ${}_{E}^{B}T_{0}$, ${}_{C}^{E}T_{0}$, and ${}_{W}^{C}P_{0}$. Rotational random noise is added to ${}_{E}^{B}T$ according to the expression $T_{ij}+e_{rot},i,j=1\cdots 3,e_{rot}∼N\left(0,\sigma_{rot}\right)$, where $e_{rot}$ obeys a Gaussian distribution $N\left(0,\sigma_{rot}\right)$. Similarly, translational random noise is added to both ${}_{E}^{B}T$ and ${}_{W}^{C}P$ according to expressions $T_{i4}+e_{tran}$ and $P_{i1}+e_{tran},i=1\cdots 3,e_{tran}∼N\left(0,\sigma_{tran}\right)$, respectively. During the simulation, $\sigma_{rot}$ and $\sigma_{tran}$ are set to 0.0001 and 0.01, respectively. The ABB IRB1410 robot model is used, and Table 1 presents the MDH parameters along with their errors (enclosed in parentheses). The camera error parameter is set to [1 × 10${}^{-9}$, 7 × 10${}^{-15}$, 5 × 10${}^{-18}$].

### 3.1. Calibration Accuracy

One hundred training data sets and one hundred testing data sets are generated. The initial ${}_{C}^{E}T$ is obtained as below. We can observe that ${}_{C}^{E}T$ deviates from ${}_{C}^{E}T_{0}$, which can be attributed to robot and camera errors.

$${}_{C}^{E}T=\left[\begin{array}{cccc} 0.0051 & -1.0000 & -0.0035 & -59.5445\\ 1.0000 & 0.0051 & -0.0034 & 61.3253\\ 0.0034 & -0.0035 & 1.0000 & 86.2298\\ 0 & 0 & 0 & 1 \end{array}\right]$$

Next, ${}_{B}^{E}T$, ${}_{C}^{E}T$, and ${}_{W}^{C}P$ are substituted into Algorithm 1 to obtain the calibration parameter *x*, and the results are presented in Table 2. The calibrated hand–eye matrix ${}_{C}^{E}T_{\mathrm{calibrated}}$ is obtained as follows.

$${}_{C}^{E}T_{\mathrm{calibrated}}=\left[\begin{array}{cccc} 0.0001 & -1.0000 & -0.0001 & -59.7917\\ 1.0000 & 0.0001 & 0.0014 & 60.0563\\ -0.0014 & -0.0001 & 1.0000 & 84.9688\\ 0 & 0 & 0 & 1 \end{array}\right]$$

It has been observed that ${}_{C}^{E}T_{\mathrm{calibrated}}$ is close to ${}_{C}^{E}T_{0}$, and $\xi_{CW}$ is close to the negative of the preset value. These results demonstrate that the joint calibration method can accurately calibrate the hand–eye matrix and camera errors. However, since $\xi_{i}\left(i=1,2,\cdots ,6\right)$ do not have true values, it is difficult to determine whether the robot error has been accurately identified. This problem will be addressed by observing the calibration result of ${}_{E}^{B}T$.

To illustrate the importance of joint calibration, the methods are classified into methods 1–4 based on whether robot, hand–eye matrix, or camera errors are taken into account, as shown in Table 3, where √ indicates that this type of error is considered and × indicates that it is not. Methods 1 and 2 utilize the error model and calibration method proposed in this paper. Method 4, proposed in Reference [26], uses the D-H method to model robot error and establishes a linearized equation for calibration. The positioning accuracy and repeatability accuracy in $\left\{B\right\}$ are chosen as the evaluation criteria to evaluate these methods. The mean value (emean) and the maximum value (emax) of $∥{}_{W}^{B}P_{\mathrm{calibrated}}-{}_{W}^{B}P_{0}∥$ calculated from 100 testing data sets are used as the evaluation criteria for positioning accuracy. The standard deviation of ${}_{W}^{B}P_{\mathrm{calibrated}}$, denoted as std, is used as the evaluation criteria for repeatability accuracy.

The results are presented in Table 3. Method 3, without calibration, exhibits a large emean, emax, and std, whereas method 1 shows significantly smaller values, indicating that the proposed joint calibration method improves both the positioning accuracy and repeatability accuracy. Method 2 shows a larger emean and emax compared to method 3 but a smaller std, suggesting that method 2 improves the repeatability accuracy while reducing positioning accuracy. Method 4 fails to effectively calibrate the compensative error.

**Figure 6:**
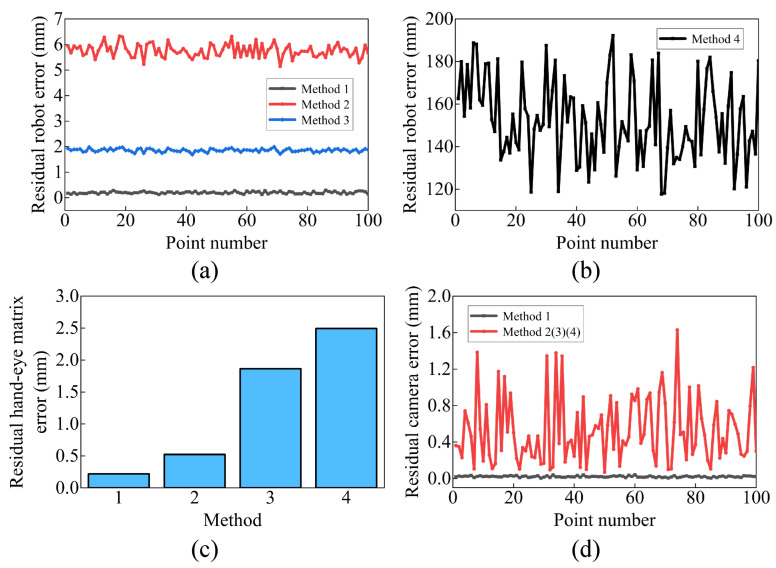
The residual error in each part: (**a**) the residual robot error in methods 1–3, (**b**) the residual robot error in method 4, (**c**) the residual error in the hand–eye matrix, (**d**) the residual error of the camera.

Notably, the proposed method’s optimization objective is to minimize the relative error in ${}_{W}^{B}P$, as shown in Equation (8). It does not guarantee the individual correct calibration of the robot, hand–eye matrix, and camera errors. ${}_{E}^{B}T_{\mathrm{calibrated}}$, ${}_{C}^{E}T_{\mathrm{calibrated}}$, and ${}_{W}^{C}P_{\mathrm{calibrated}}$ are compared with their ideal values to observe the calibration results of the robot, hand–eye matrix, and camera errors separately. $∥P_{\mathrm{calibrated}}-P_{0}∥$ and $∥{T_{\mathrm{calibrated}}}_{\left(1:4,4\right)}-{T_{0}}_{\left(1:4,4\right)}∥$ are chosen as the evaluation criteria. Figure 6 displays the calibration results of each testing data. Method 1 achieves great calibration for all three types of error. Method 2 only calibrates partly the hand–eye matrix error, whereas method 4 is less effective in calibrating each error.

### 3.2. The Impact of Random Noise

**Figure 7:**
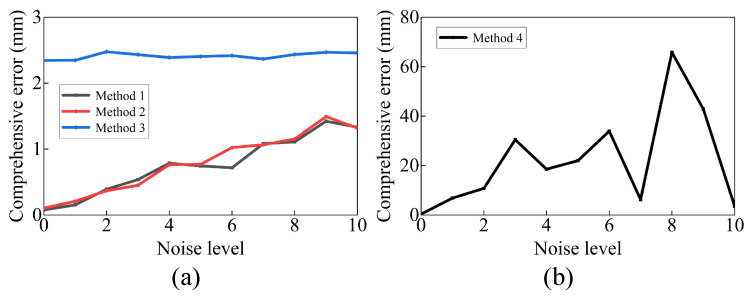
The impact of random noise on each method: (**a**) methods 1–3, (**b**) method 4.

To investigate the impact of random noise on each method, we gradually increase the random noise by multiplying a coefficient “noise level” to $\sigma_{rot}$ and $\sigma_{tran}$, while setting $\xi_{CW}$ to 0. The variation in emean is observed and presented in Figure 7. Even though emean increases with the noise level, method 1 consistently produces smaller emean than method 3. This indicates that method 1 is able to resist noise and maintain accuracy. Method 2 also achieves a similar result since the camera error is set to 0. Method 4 can calibrate the comprehensive error only when the random noise is small, indicating its high sensitivity to random noise.

### 3.3. The Impact of Camera Error

**Figure 8:**
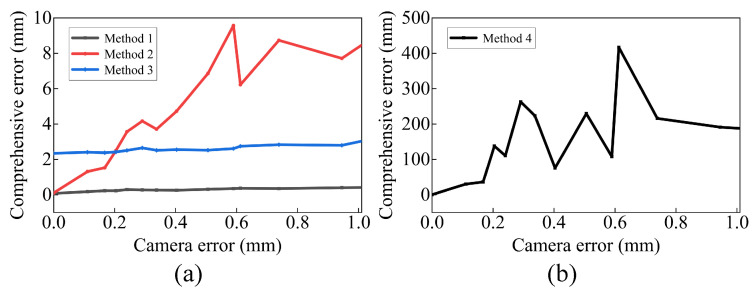
The impact of camera error on each method: (**a**) methods 1–3, (**b**) method 4.

To investigate the impact of camera error on each method, we gradually increase the preset value of $\xi_{CW}$ while setting the noise level to 0, and obtain the variation in emean for each method. The results are presented in Figure 8, where the horizontal axis represents the mean error in ${}_{W}^{C}P$. We observe that as the camera error increases, the emean of method 1 gradually increases but remains smaller than that of method 3. Similarly, the emean of method 2 also increases with the camera error. However, method 2 exhibits a negative effect when the camera error exceeds 0.2 mm, indicating the need to consider camera error. Method 4 can yield a small emean only when the camera error is small, indicating its high sensitivity to camera error.

## 4. Experiment

**Figure 9:**
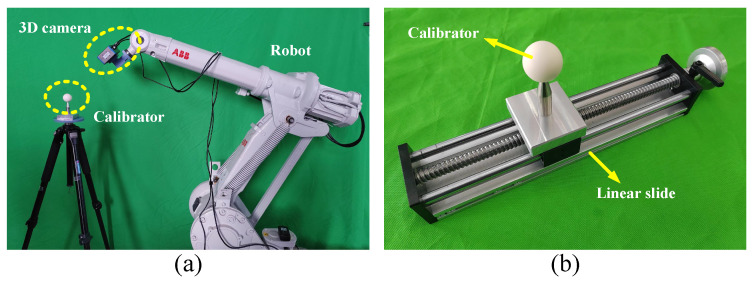
Experimental equipment: (**a**) the calibration system, (**b**) the standard sphere installed on a linear slide.

The calibration system, as shown in Figure 9a, utilizes an ABB IRB1410 robot with a repeatability accuracy of ±0.05 mm. The parameters of the camera and ceramic standard sphere are detailed in Section 2.2.

### 4.1. Calibration Accuracy

The initial ${}_{C}^{E}T$ is obtained as follows.

$${}_{C}^{E}T=\left[\begin{array}{cccc} 0.0135 & -0.9998 & 0.0109 & -57.4017\\ 0.9995 & 0.0132 & -0.0278 & 61.1012\\ 0.0276 & 0.0113 & 0.9996 & 86.1401\\ 0 & 0 & 0 & 1 \end{array}\right]$$

Next, ${}_{B}^{E}T$, ${}_{C}^{E}T$, and ${}_{W}^{C}P$ are substituted into Algorithm 1 to solve for *x*, and the results are presented in Table 4. The calibrated hand–eye matrix is obtained as follows.

$${}_{C}^{E}T_{\mathrm{calibrated}}=\left[\begin{array}{cccc} -0.0023 & -0.9999 & -0.0010 & -57.2041\\ 1.0000 & -0.0023 & -0.0032 & 60.9219\\ 0.0032 & -0.0010 & 1.0000 & 86.5221\\ 0 & 0 & 0 & 1 \end{array}\right]$$

To evaluate the performance of methods 1–4, distance accuracy is used instead of positioning accuracy, since ${}_{W}^{B}P_{0}$ is unknown in the experiment. The distance accuracy is tested as follows. After obtaining the calibration parameters using each method, the standard sphere is fixed on a linear slide table with a distance accuracy of ±0.03 mm, as shown in Figure 8b. ${}_{W}^{C}P_{1}$ is obtained from 25 different poses and then converted to ${}_{W}^{B}P_{1}$ using the calibration parameters. The linear slide is then moved by 50 mm, and the process is repeated to obtain ${}_{W}^{B}P_{2}$. The mean value (demean) and the maximum value (demax) of $\mathrm{abs}\left({∥{}_{W}^{B}P_{1}-{}_{W}^{B}P_{2}∥}_{2}-50\right)$ calculated from 25 data sets are used as evaluation criteria for the distance accuracy, and $\mathrm{std}\left(\mathrm{abs}\left({∥{}_{W}^{B}P_{1}-{}_{W}^{B}P_{2}∥}_{2}-50\right)\right)$ (dstd) is used as the evaluation criterion for repeatability accuracy. The results obtained are shown in Table 5.

Method 3 exhibits the largest demean, demax, and dstd compared to other methods, whereas method 1 yields the smallest. This result suggests that the proposed joint calibration method performs exceptionally well in practical situations. Although method 2 shows slightly larger values than method 1, it still clearly outperforms method 3. In addition, method 2 could partially calibrate comprehensive error, indicating that camera error is insignificant. Method 4 also performs calibration successfully, unlike the result in Table 3. Method 4 disregards the camera error and relies on the D-H method, making it sensitive to the camera error. When the camera error is significant, method 4 will fail to calibrate the measurement system. To show this case, a significant camera error is set in the simulation to exhibit the necessity of considering the camera error and modeling using Lie algebra. As shown in Figure 6d, a maximum of 1.6 mm camera error is added in the simulation, and method 4 fails to achieve calibration. Figure 8b demonstrates that method 4 can also perform high-precision calibration with a small camera error. The maximum fitted diameter error is less than 0.4 mm in the experiment, as shown in Figure 5. The error is small enough that method 4 can successfully calibrate the measurement system. The experimental results actually align with the simulation results. While the proposed method’s advantage may not be as pronounced as in the simulations, the results in Table 5 indicate a notable improvement over other methods.

**Figure 10:**
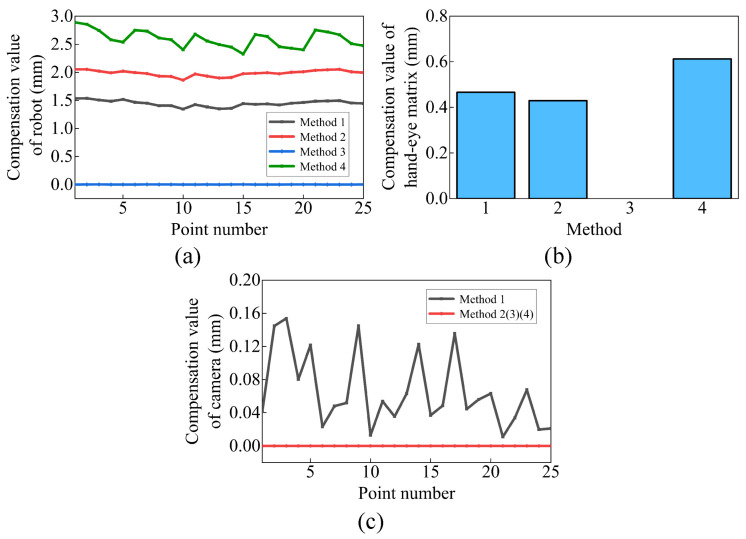
The compensation value of each part: (**a**) robot, (**b**) hand–eye matrix, and (**c**) camera.

The individual compensation values for the robot, hand–eye matrix, and camera errors are shown in Figure 10. It is important to note that the compensation value can only be used as a reference for each error. We can infer from Figure 10 that robot error is the main error, followed by hand–eye matrix error, and camera error is the smallest. This result is consistent with the previous inference.

**Figure 11:**
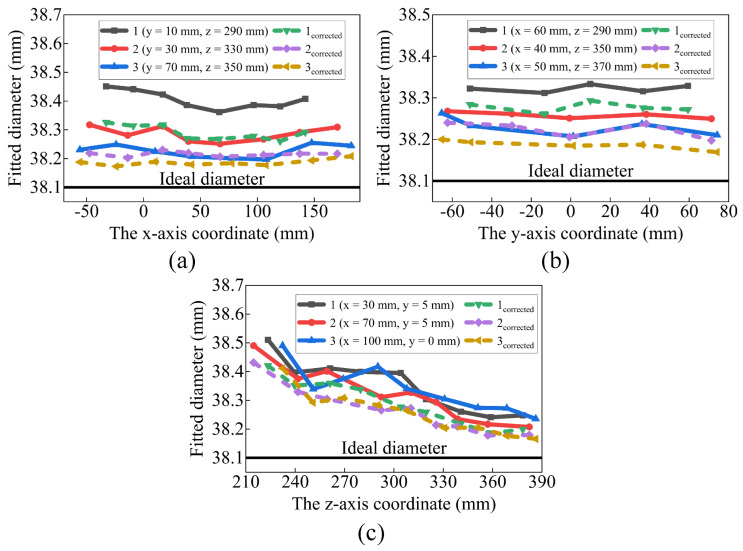
The calibration results of the camera (**a**) along the x-axis direction, (**b**) along the y-axis direction, (**c**) along the z-axis direction.

The $\xi_{CW}$ obtained from method 1 is used to calibrate the point clouds in Figure 5, and the results are shown in Figure 11. The fitted diameter after calibration is closer to the theoretical diameter, indicating that the joint calibration method successfully calibrates part of the camera error. However, the diameter deviation is not completely eliminated, which could be attributed to two reasons. Firstly, during the joint calibration method, only the sphere center and not all point clouds of the sphere are used, which can lead to the deviation in $\xi_{CW}$. Secondly, the camera may have other error forms that are not accounted for.

### 4.2. Performance in Practical Applications

**Figure 12:**
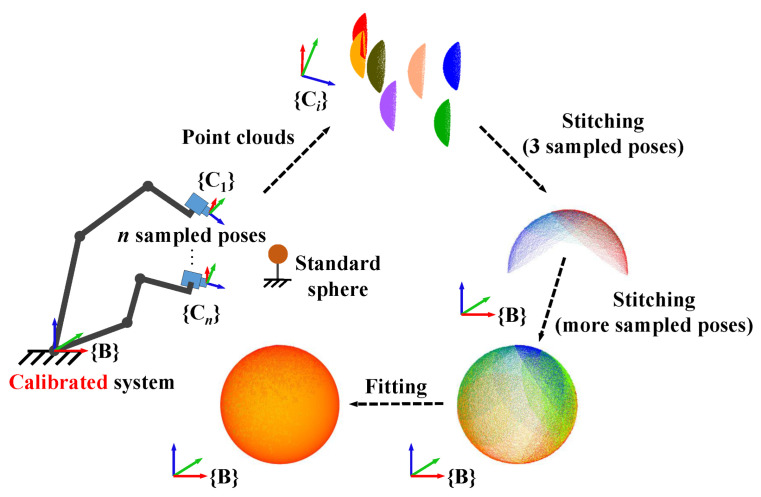
The point clouds stitching and spherical fitting process.

**Figure 13:**
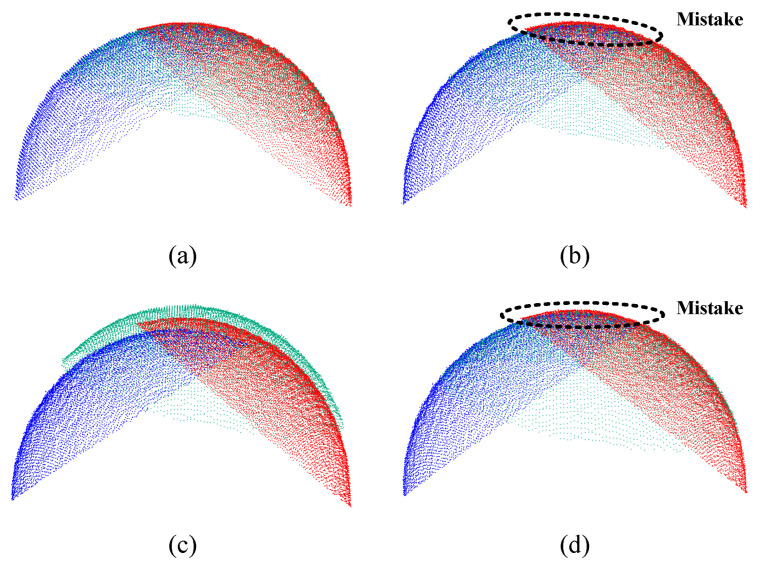
Stitching results of three point clouds using each method: (**a**) method l, (**b**) method 2, (**c**) method 3, and (**d**) method 4.

The robot measurement system is mainly calibrated to measure workpieces precisely. To evaluate each method in practical measurements, the calibrated robot measurement system is used to measure the standard sphere, which is placed at another position, different from that during the calibration process. Multiple point clouds of the standard sphere are collected from different poses and stitched together, and the performance of each method is assessed based on the stitching quality. For better visualization, only three point clouds are stitched first, and the stitching process and results are presented in the upper parts of Figure 12 and Figure 13, respectively. Method 1 produces the best stitching result, whereas methods 2 and 4 exhibit more mistakes than method 1. Method 3 yields the worst stitching result, with misaligned point clouds. These results suggest that the joint calibration method significantly improves measurement accuracy in practice.

**Figure 14:**
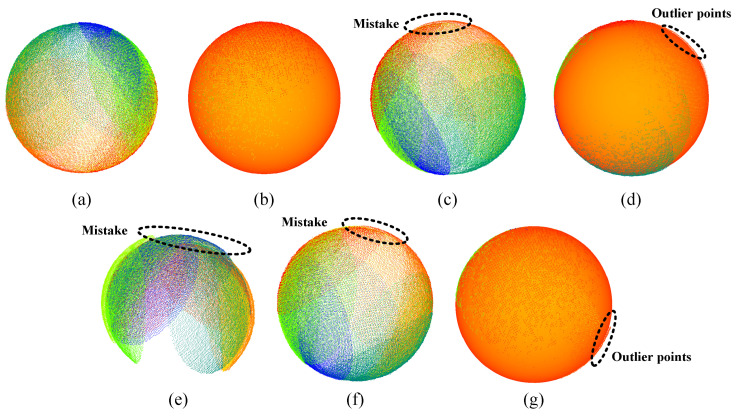
Stitching results and fitting results of each method: (**a**) the stitching result of method 1, (**b**) the fitting result of (**a**), (**c**) the stitching result of method 2, (**d**) the fitting result of (**c**), (**e**) the stitching result of method 3, (**f**) the stitching result of method 4, (**g**) the fitting result of (**f**).

After that, point clouds from more sampled poses are stitched together and fitted to spheres, as shown in the bottom part of Figure 12. The stitching and spherical fitting results of multiple point clouds are shown in Figure 14. Method 1 also yields the highest-quality stitching result. Methods 2 and 4 exhibit noticeable mistakes, with method 4 failing to stitch the point clouds. In terms of fitting results, method 1 outperforms the other methods, whereas methods 2 and 4 feature numerous outlier points. The error between the fitted diameter and theoretical value is used to evaluate each method quantitatively. The fitted diameter errors of methods 1, 2, and 4 are 0.23 mm, 0.61 mm, and 0.57 mm, respectively, whereas method 4 fails to achieve a proper spherical fitting. The result also demonstrates that method 1 exhibits the highest calibration accuracy.

## 5. Conclusions

This paper proposes a joint calibration method for a robot measurement system, which considers robot kinematic, camera-to-robot installation, and 3D camera measurement errors. This method establishes separate error models for each type of error and constructs a joint error model based on homogeneous transformation. Based on the joint error model, the calibration problem is formulated as a stepwise optimization problem, and analytical solutions are derived. The superiority of the proposed method is validated through simulation and experiment. In the simulation, the proposed method can reduce the mean positioning error from over 2.5228 mm to 0.2629 mm and the mean repeatability error from over 0.1831 mm to 0.0112 mm, compared to methods without considering all error types. In addition, the anti-noise simulation results demonstrate that the proposed method can achieve reliable high-precision calibration, even in increasing random noise. In the experiment, the proposed method can reduce the mean distance error from over 0.1488 mm to 0.1232 mm and the mean repeatability error from over 0.1045 mm to 0.0957 mm compared to other methods. When applied to actual measurements, the proposed method outperforms other methods in stitching and fitting point clouds, reducing the fitted diameter error from over 0.57 mm to 0.23 mm. However, based on the experimental results, the proposed method can only partially calibrate the 3D camera measurement error. Other forms of 3D camera measurement error, except for that proposed in Section 2.2, may exist and require further investigation.

## Figures and Tables

**Table 1:** The parameters and errors of the robot.

Joint Number	$a_{j-1}$ (mm)	$\alpha_{j-1}$ (rad)	$d_{j}$ (mm)	$\theta_{j}$ (rad)
1	0 (0.0016)	0 (1.5 × 10${}^{-4}$)	475 (0.0018)	0 (3.0 × 10${}^{-4}$)
2	150 (0.0023)	0.5π (7.5 × 10${}^{-4}$)	0 (0.0033)	0.5π (4.5 × 10${}^{-4}$)
3	600 (0.0390)	0 (3.0 × 10${}^{-4}$)	0 (0.0071)	0 (−1.5 × 10${}^{-4}$)
4	120 (−0.030)	0.5π (9.0 × 10${}^{-4}$)	720 (0.0075)	0 (3.0 × 10${}^{-4}$)
5	0 (0.0240)	−0.5π (7.5 × 10${}^{-4}$)	0 (0.0032)	0 (−0.0011)
6	0 (−0.0510)	0.5π (−4.5 × 10${}^{-4}$)	85 (0.0018)	0 (1.5 × 10${}^{-4}$)

**Table 2:** The simulation results of calibration parameters.

	$\rho_{1}$ (mm)	$\rho_{2}$ (mm)	$\rho_{3}$ (mm)	$\phi_{1}$ (rad)	$\phi_{2}$ (rad)	$\phi_{3}$ (rad)
$\xi_{1}$	−0.0386	0.0141	−0.0320	0.0250	0.0505	−0.2077
$\xi_{2}$	−0.4040	0.4460	0.5121	−0.4951	−0.0021	0.0545
$\xi_{3}$	5.62 × 10${}^{-4}$	−0.1397	−0.1761	0.4880	0.5026	−0.0640
$\xi_{4}$	−9.91 × 10${}^{-6}$	−3.04 × 10${}^{-4}$	−8.92 × 10${}^{-4}$	−7.50 × 10${}^{-4}$	−4.54 × 10${}^{-4}$	−0.0014
$\xi_{5}$	−8.97 × 10${}^{-6}$	−4.84 × 10${}^{-6}$	4.42 × 10${}^{-4}$	8.53 × 10${}^{-4}$	0.0034	−1.31 × 10${}^{-4}$
$\xi_{6}$	8.75 × 10${}^{-4}$	3.72 × 10${}^{-4}$	−6.04 × 10${}^{-4}$	1.24 × 10${}^{-4}$	1.98 × 10${}^{-4}$	0.0031
$\xi_{EC}$	−1.2709	0.2462	−1.2593	0.0033	0.0048	0.0049
	$k_{1}$	$k_{2}$	$k_{3}$			
$\xi_{CW}$	−9.60 × 10${}^{-10}$	−7.32 × 10${}^{-15}$	−4.94 × 10${}^{-18}$			

**Table 3:** Positioning and repeatability errors of methods.

Method	Robot	Hand–Eye Matrix	Camera	emean (mm)	emax (mm)	std (mm)
1	√	√	√	0.2629	0.2918	0.0112
2	√	√	×	5.6545	5.9715	0.1831
3	×	×	×	2.5228	3.5615	0.5317
4	√	√	×	134.8228	140.9125	0.6630

**Table 4:** The experimental results of the calibration parameters.

	$\rho_{1}$ (mm)	$\rho_{2}$ (mm)	$\rho_{3}$ (mm)	$\phi_{1}$ (rad)	$\phi_{2}$ (rad)	$\phi_{3}$ (rad)
$\xi_{1}$	0.1416	−0.2921	−1.35 × 10${}^{-4}$	−1.56 × 10${}^{-4}$	6.66 × 10${}^{-4}$	3.26 × 10${}^{-5}$
$\xi_{2}$	0.5372	−0.1758	−0.0512	2.32 × 10${}^{-4}$	−1.58 × 10${}^{-4}$	4.73 × 10${}^{-5}$
$\xi_{3}$	0.3549	0.1113	−0.0251	2.46 × 10${}^{-4}$	−1.28 × 10${}^{-4}$	1.25 × 10${}^{-6}$
$\xi_{4}$	−0.1963	0.2188	0.6091	4.84 × 10${}^{-4}$	−5.40 × 10${}^{-4}$	9.96 × 10${}^{-5}$
$\xi_{5}$	0.1579	−0.0294	−0.2141	8.81 × 10${}^{-5}$	6.02 × 10${}^{-4}$	1.73 × 10${}^{-4}$
$\xi_{6}$	0.0977	0.0743	0.0969	−1.78 × 10${}^{-4}$	3.44 × 10${}^{-5}$	4.60 × 10${}^{-7}$
$\xi_{EC}$	−0.1723	−0.1967	0.3857	−0.0121	0.0245	0.0157
	$k_{1}$	$k_{2}$	$k_{3}$			
$\xi_{CW}$	7.02 × 10${}^{-28}$	4.32 × 10${}^{-23}$	7.58 × 10${}^{-18}$			

**Table 5:** Distance and repeatability errors of methods.

Method	Robot	Hand–Eye Matrix	Camera	demean (mm)	demax (mm)	dstd (mm)
1	√	√	√	0.1232	0.3137	0.0957
2	√	√	×	0.1488	0.3675	0.1045
3	×	×	×	1.5457	4.6076	1.3503
4	√	√	×	0.1933	0.4703	0.1414

## Data Availability

The data presented in this study are available on request from the corresponding author.
